# Correlation between vascular adhesion protein-1 and major adverse cardiovascular events in patients with atrial fibrillation

**DOI:** 10.3389/fcvm.2025.1684589

**Published:** 2025-11-21

**Authors:** You Zhang, Chi Geng, Feng Li, Yulun Zhou, Siliang Peng, Mengchao Jin, Xinru Guo, Zhiyuan Zhang, Xiaosong Gu, Jing Li, Hui Li

**Affiliations:** 1 Department of Cardiology, The Second Affiliated Hospital of Soochow University, Suzhou, Jiangsu, China; 2 Department of Intensive Care Medicine, The First Affiliated Hospital of Soochow University, Suzhou, Jiangsu, China

**Keywords:** vascular adhesion protein-1, atrial fibrillation, major adverse cardiovascular events, inflammation, oxidative stress

## Abstract

**Background:**

VAP-1, an inflammation-induced endothelial molecule, is implicated in cardiovascular diseases, but its role in AF is unclear. This study aimed to explore the relationship between serum VAP-1 levels and AF risk, as well as its prognostic significance.

**Methods:**

We retrospectively analyzed the clinical data of 356 hospitalized patients at the Second Affiliated Hospital of Soochow University from May 2020 to September 2022, of whom 99 were diagnosed with AF. Serum VAP-1 levels were measured using enzyme-linked immunosorbent assay (ELISA) at enrollment. Associations between AF onset and VAP-1 levels were assessed. The primary endpoint was the occurrence of major adverse cardiovascular events (MACE). Clinical data were obtained from electronic medical records and telephone follow-ups. Regression analysis, curve fitting, and survival analysis were used to evaluate these associations.

**Results:**

Retrospective analysis and curve fitting revealed an association between elevated VAP-1 levels and the onset of AF (HR = 1.001, 95% CI = 1.000–1.002). After adjustment for possible confounding factors, higher serum VAP-1 levels were associated with an increased risk of MACE in patients with AF (HR = 5.28, 95% CI = 0.64–43.66) and (HR = 28.35, 95% CI = 2.82–284.92). The results obtained from curve fitting and survival analysis corroborated the findings of the prior regression analysis.

**Conclusion:**

The results revealed a significant correlation between elevated VAP-1 levels and both the incidence of AF and the occurrence of MACE, suggesting that VAP-1 may serve as a valuable biomarker for predicting the onset and prognosis of AF.

## 1 Introduction

Atrial fibrillation (AF) is the most common cardiac arrhythmia and can significantly increase the risk of major adverse cardiovascular events (MACE, including heart attack, heart failure, stroke and death), cognitive dysfunction and dementia, exerting a serious impact on the health and quality of patients. The incidence of AF rises with age, as the population aged, more burden will fall on health care delivery systems. The pathogenesis of AF is intricate and multifactorial, encompassing age, primary cardiac conditions (e.g., coronary artery disease, cardiomyopathy), non-cardiac comorbidities (e.g., hyperthyroidism, diabetes mellitus), unhealthy lifestyle habits, genetic predispositions, and other factors (1). The latest U.S. guideline modified the classifications of AF, dividing it into different stages, emphasizing that AF is a progressive disease that requires different strategies at the different stages (2). Despite the improving of risk stratification, population screening, and advances in management strategies such as stroke prevention, rhythm control and catheter ablation, a significant number of patients are still suffering from AF or its complications. Therefore, the exploration of novel diagnostic or prognostic biomarkers for AF is clinically valuable, as it may improve clinical management.

The inflammatory response and oxidative stress are among the key pathological features and underlying pathogenic mechanisms of atrial fibrillation. Vascular adhesion protein 1 (VAP-1), acting as an inflammatory mediator, is present in both membrane-bound and soluble forms. Membrane-bound VAP-1 has a broad tissue distribution, it expresses on different cell types such as endothelial cells, smooth muscle cells, leukocytes and adipocytes. And it can be cleaved by matrix metalloproteinases (MMPs) and released as soluble forms (3). Functionally, VAP-1 can serve as an adhesion molecule, regulating the migration of leukocytes across vascular endothelial cells and tissues, thereby facilitating the inflammatory response. VAP-1, also referred to as semicarbazide-sensitive amine oxidase (SSAO), is capable of catalyzing the oxidative deamination of primary amines, its catalytic byproduces, such as formaldehyde/glyoxal, ammonia, and H_2_O_2_, can induce cross-linking of proteins and/or DNA, as well as oxidative stress (3). Therefore, VAP-1 is implicated in a variety of physiological and pathological processes and has emerged as a potential biomarker for a broad spectrum of inflammatory-related diseases across different systems, including pulmonary diseases [e.g., chronic obstructive pulmonary disease (4), respiratory tract allergic diseases (5)], cardiovascular diseases [e.g., heart failure (6), atherosclerosis (7), coronary heart disease (8)], digestive diseases [e.g., chronic liver disease (9), inflammatory bowel disease (10)], chronic kidney disease (11), tumors (12, 13), obesity (14), diabetes and diabetic complications (15, 16), neurological disorders [e.g., Alzheimer's disease (17)], etc.

Considerable evidence has shown that VAP-1 is linked to several common cardiovascular diseases (CVD) and is associated with the cardiovascular prognosis of some inflammatory and metabolic diseases. Elevated serum levels of VAP-1 were found in atherosclerotic cardiovascular diseases (7), chronic heart failure (6), and stroke (18). Higher VAP-1 levels have also been demonstrated to be associated with an increased risk of MACE in diverse populations, including patients with heart failure (19), hemodialysis (20), T2DM (21), and individuals over 50 years of age who have not previously experienced (22). Our previous cohort study found that higher levels of soluble VAP-1 were closely related to the increased occurrence of MACE in patients with coronary heart disease (CHD) (8), the pathogenic role of VAP-1 in atherosclerotic diseases and its potential as a therapeutic target have also been investigated in several basic research studies (7).

Therefore, VAP-1 is a noteworthy cardiovascular disease-related inflammatory factor, but as of yet, no research has delved into the connection between VAP-1 and AF. The present study was designed to explore the association between VAP-1 and the risk of AF, along with its prognostic implications.

## 2 Methods

### 2.1 Study design and participants

**Figure 1:**
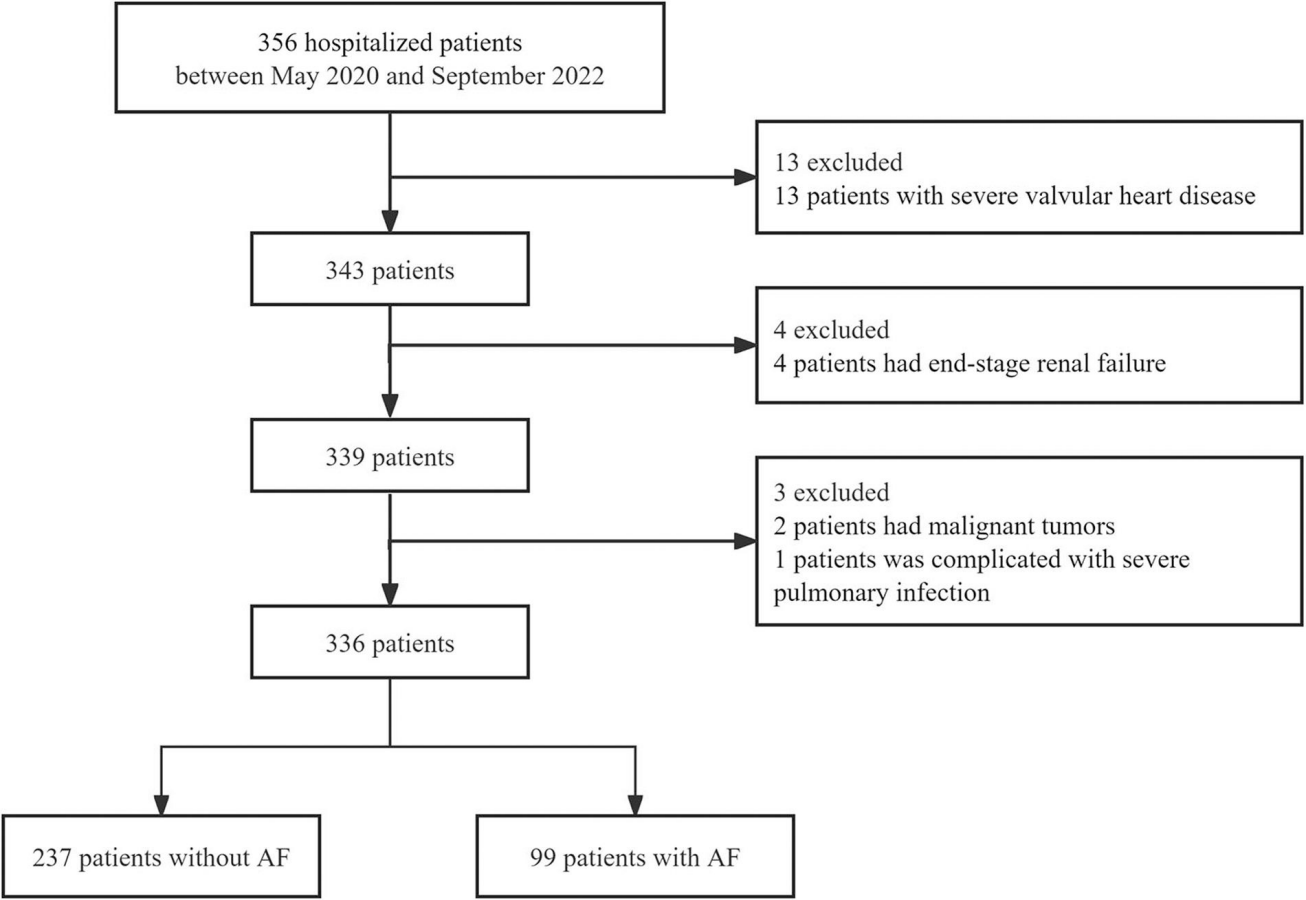
Flowchart of participant selection. VAP-1, vascular adhesion protein-1; AF, atrial fibrillation.

This retrospective cohort study included 356 patients admitted to the Department of Cardiology at the Second Affiliated Hospital of Soochow University from May 2020 to September 2022. Inclusion criteria were: (1) age ≥18 years; (2) Patients with atrial fibrillation were diagnosed via electrocardiogram or Holter monitoring during or prior to hospitalization (including all types of atrial fibrillation), while non-atrial fibrillation patients were diagnosed with normal sinus rhythm and were not detected with atrial fibrillation or atrial flutter. Exclusion criteria included moderate/severe mitral or aortic valve disease, end-stage renal failure, malignancies, and severe pulmonary infections (Figure 1). A total of 336 patients were ultimately included in the analysis, of whom 99 had atrial fibrillation. The study was approved by the ethics committee of the Second Affiliated Hospital of Soochow University (JD-LK-2022-125-01) and adhered to the Helsinki Declaration (2013 revision).

### 2.2 Measurement of plasma VAP-1 concentration

Plasma samples for VAP-1 testing were collected from fasting patients during admission and stored at −80 °C. Written informed consent was obtained from all participants. VAP-1 levels were measured using the VAP-1 Human ELISA Kit (BMS259TEN, Invitrogen, USA) via a solid-phase sandwich ELISA method. Briefly, target antibodies were precoated on a microplate, samples were added, and a streptavidin-horseradish peroxidase complex was formed. A substrate solution was then added to initiate a colorimetric reaction, producing a signal proportional to VAP-1 concentration. Optical density was measured at 450/630 nm using a BioTek ELx800 microplate reader to calculate VAP-1 levels.

### 2.3 Covariates

Demographic data, comorbidities, personal history, vital signs, and laboratory results were collected from medical records or follow-up calls. Comorbidities were diagnosed based on clinical guidelines or medical history. For AF patients, the CHA2DS2-VASc score was used to assess thromboembolism risk. Standard laboratory methods were employed to measure a series of parameters, which included routine hematological indicators of liver and kidney function, blood count and lipid profile, C-reactive protein (CRP), fasting blood glucose (FBG), glycated hemoglobin A1c (HbA1c), creatine kinase (CK), cardiac troponin T (CTnT), N-terminal brain natriuretic peptide precursor (NT-proBNP) and D-dimer. The echocardiographic evaluation of cardiac structure and function was conducted with Model GE Vivid E9, using an M5S phased array transducer with a transmission frequency of 2.0–4.5 MHz. The parameters of cardiac structure such as the left atrial diameter (LAD) and the left ventricular end-diastolic dimension (LVDD) were assessed and recorded. The left ventricular ejection fraction (LVEF) was calculated using the modified Simpson method from the apical 4- and 2-chamber views (23). All measurements were obtained by an experienced echocardiographer.

### 2.4 Follow-up

Patients were followed-up regularly for a median duration of 30 months (interquartile range: 17–35 months) in the outpatient ward or by telephone. The primary outcome was the occurrence of MACE, defined as a composite of cardiovascular death, myocardial infarction, non-myocardial infarction acute coronary syndrome, stroke or acute decompensated HF.

### 2.5 Statistical analysis

Categorical variables were presented as counts and percentages, while normally distributed continuous variables were shown as mean ± SD, and non-normally distributed variables as median (IQR). Chi-square tests were used for categorical variables, rank sum tests for non-normally distributed continuous variables, and one-way ANOVA for normally distributed continuous variables. Multivariate Cox regression analysis was performed to assess the independent association of VAP-1 with MACE in all patients and in those with AF, using both non-adjusted and adjusted models. Covariates were selected based on clinical judgment and included in models if significant in univariate analysis or causing >10% change in initial regression coefficients. Model I adjusted for age and gender; Model II for age, gender, and other significant covariates; and Model III considered statistical significance and clinical relevance. The association between VAP-1 and MACE was depicted using smooth curves, and Kaplan–Meier analysis with log-rank tests was used to compare event rates. Missing values (<6%) in continuous variables were imputed with median or mean values, with no significant differences in baseline characteristics before and after imputation (Supplementary Table S1). Data were analyzed using Free Statistics software version 2.0 and R 4.4 (http://www.R-project.org). A two-sided *P* < 0.05 was considered significant.

## 3 Results

### 3.1 Characteristics of the study population

Of the 356 participants, we excluded 13 patients with severe valvular heart disease, 4 with end-stage renal failure at baseline, and 3 with cancer or severe lung diseases (Figure 1). Ultimately, 336 patients were included in the study, of whom 99 were diagnosed with atrial fibrillation on the basis of their medical history or electrocardiographic (ECG) findings (Figure 1).

All AF patients were stratified into 3 groups according to the tertiles of VAP-1, their baseline characteristics are presented in Table 1. At baseline, the mean age of these patients with AF was 72.3 ± 9.3 years, and 68.7% of them were male. The results showed that the proportion of HF and the NT-proBNP level were positively related to the level of VAP-1 in AF patients (*P* = 0.003), whereas the LVEF had an inverse association with VAP-1 (*P* = 0.001), the renal function indicators Scr and eGFR were positively and negatively correlated, respectively, to VAP-1 levels in AF patients (*P* = 0.004, *P* < 0.001). Notably, during a median follow-up of 30 months (IQR, 17–35), the time to MACE was significantly shorter in AF patients with higher VAP-1 levels (*P* < 0.001).

**Table 1:** Baseline characteristics grouped according to VAP-1 level in AF patients.

Variables	VAP-1(ng/mL)				*P* value
	Total	Q1(≤895)	Q2 (895–1,450)	Q3 (≥1,450)	
	(*n* = 99)	(*n* = 33)	(*n* = 33)	(*n* = 33)	
AF types, *n* (%)					0.877
PAF	36 (36.4)	13 (39.4)	12 (36.4)	11 (33.3)	
PeAF	63 (63.6)	20 (60.6)	21 (63.6)	22 (66.7)	
Gender (male), *n* (%)	68 (68.7)	19 (57.6)	24 (72.7)	25 (75.8)	0.233
Age (years)	72.3 ± 9.3	71.1 ± 10.9	73.1 ± 8.4	72.7 ± 8.4	0.666
BMI (Kg/m^2^)	24.2 ± 3.8	25.4 ± 4.3	23.6 ± 3.3	23.6 ± 3.7	0.081
HF, *n* (%)	76 (76.8)	26 (78.8)	20 (60.6)	30 (90.9)	0.014
IS, *n* (%)	15 (15.2)	4 (12.1)	4 (12.1)	7 (21.2)	0.493
CHD, *n* (%)	32 (32.3)	10 (30.3)	11 (33.3)	11 (33.3)	0.955
HBP, *n* (%)	67 (67.7)	22 (66.7)	23 (69.7)	22 (66.7)	0.955
DM, *n* (%)	26 (26.3)	8 (24.2)	8 (24.2)	10 (30.3)	0.812
COPD, *n* (%)	10 (10.1)	4 (12.1)	1 (3)	5 (15.2)	0.35
Smoke, *n* (%)	29 (29.3)	12 (36.4)	10 (30.3)	7 (21.2)	0.396
Drink, *n* (%)	11 (11.1)	6 (18.2)	3 (9.1)	2 (6.1)	0.386
CHA2DS2-VASc, (score)	3.8 ± 1.7	3.7 ± 1.8	3.7 ± 1.9	4.1 ± 1.5	0.638
SBP (mmHg)	133.4 ± 21.8	137.2 ± 18.2	129.6 ± 20.4	133.3 ± 26.1	0.367
DBP (mmHg)	76.9 ± 14.1	78.9 ± 11.6	77.1 ± 14.1	74.8 ± 16.4	0.498
HR (beats per min)	84.0 ± 16.0	85.2 ± 17.3	84.2 ± 16.4	82.6 ± 14.7	0.824
WBC (×10^9^/L)	6.7 ± 2.5	6.7 ± 2.6	7.2 ± 2.9	6.2 ± 2.0	0.291
Neutrophil (×10^9^/L)	4.8 ± 2.4	4.9 ± 2.6	5.2 ± 2.7	4.3 ± 1.8	0.267
Lymphocyte (×10^9^/L)	1.3 ± 0.5	1.3 ± 0.6	1.3 ± 0.5	1.3 ± 0.4	0.899
Monocyte (×10^9^/L)	0.5 ± 0.2	0.4 ± 0.1	0.5 ± 0.2	0.4 ± 0.2	0.387
Hb (g/L)	129.3 ± 19.4	131.0 ± 17.3	126.8 ± 20.7	130.2 ± 20.3	0.66
PLT (×1,012/L)	175.0 ± 61.2	183.1 ± 62.6	183.0 ± 63.2	158.7 ± 56.0	0.183
CRP (mg/L)	5.9 ± 2.4	5.6 ± 2.4	5.5 ± 2.0	6.5 ± 2.8	0.205
Scr (umol/L)	89.4 ± 32.2	76.5 ± 20.2	89.9 ± 38.5	102.9 ± 30.7	0.004
Bun (mmol/L)	7.0 ± 2.3	6.3 ± 2.2	7.3 ± 2.2	7.5 ± 2.4	0.106
UA (umol/L)	425.6 ± 146.8	425.2 ± 113.7	393.6 ± 162.7	459.2 ± 156.6	0.199
eGFR (ml/min)	76.8 ± 29.2	89.0 ± 26.3	79.9 ± 32.0	61.5 ± 22.0	<0.001
TG (mmol/L)	1.1 ± 0.5	1.3 ± 0.6	1.0 ± 0.5	1.0 ± 0.5	0.094
TC (mmol/L)	3.5 ± 0.9	3.7 ± 1.0	3.5 ± 0.9	3.3 ± 0.9	0.244
HDL (mmol/L)	1.1 ± 0.3	1.1 ± 0.3	1.1 ± 0.2	1.0 ± 0.3	0.039
VLDL (mmol/L)	0.4 ± 0.3	0.5 ± 0.4	0.4 ± 0.3	0.4 ± 0.2	0.716
LDL (mmol/L)	2.1 ± 0.9	2.2 ± 1.0	2.3 ± 1.0	1.9 ± 0.8	0.166
FBG (mmol/L)	6.2 ± 2.0	5.7 ± 1.3	6.1 ± 1.6	6.6 ± 2.6	0.164
HbA1c (%)	6.2 ± 0.8	5.9 ± 0.5	6.3 ± 0.9	6.3 ± 0.9	0.041
ALT (U/L)	25.0 ± 20.1	21.6 ± 18.1	24.8 ± 19.9	28.3 ± 22.2	0.409
AST (U/L)	31.3 ± 35.7	31.6 ± 43.4	35.3 ± 41.6	27.1 ± 16.2	0.649
TBIL (mmol/L)	15.3 ± 7.8	15.0 ± 5.7	14.2 ± 8.8	16.6 ± 8.7	0.458
IBIL (mmol/L)	6.4 ± 3.0	5.8 ± 2.1	6.2 ± 3.3	7.4 ± 3.4	0.102
ALB (g/L)	37.7 ± 4.4	38.0 ± 4.1	38.0 ± 4.5	37.1 ± 4.5	0.63
Sodium (mmol/L)	140.7 ± 4.1	141.2 ± 3.6	141.3 ± 4.4	139.6 ± 4.3	0.181
Potassium (mmol/L)	3.9 ± 0.5	3.9 ± 0.4	4.1 ± 0.6	3.9 ± 0.5	0.278
Chlorine (mmol/L)	103.7 ± 4.5	104.2 ± 3.4	103.3 ± 4.1	103.6 ± 5.8	0.721
CK (U/L)	168.6 ± 395.3	158.0 ± 378.7	242.1 ± 566.2	105.5 ± 68.9	0.371
CTnT (pg/mL)	226.8 ± 829.7	229.5 ± 817.4	407.9 ± 1,167.4	43.1 ± 89.0	0.204
NT-proBNP (pg/mL)	5,689.2 ± 7,760.9	2,880.5 ± 3,059.2	4,992.3 ± 7,668.8	9,173.8 ± 9,752.0	0.003
D dimer (ug/mL)	1.2 ± 1.7	0.8 ± 0.9	1.3 ± 1.6	1.5 ± 2.2	0.238
LVEF (%)	49.8 ± 15.2	56.1 ± 12.5	50.5 ± 14.4	42.9 ± 16.0	0.001
LAD (mm)	50.8 ± 8.5	52.0 ± 7.7	48.8 ± 9.8	51.7 ± 7.8	0.258
LVDD (mm)	53.6 ± 8.9	51.5 ± 7.0	52.8 ± 10.0	56.5 ± 9.0	0.065
MACE, *n* (%)	39 (39.4)	13 (39.4)	10 (30.3)	16 (48.5)	0.319
Time of MACE (weeks)	17.8 ± 10.8	23.7 ± 10.7	19.3 ± 10.1	10.3 ± 6.4	<0.001

Percentage calculated from the total population; some factors total <100% due to missing data.

CHA2DS2-VASc is a scoring system for stroke risk assessment in patients with atrial fibrillation (congestive heart failure, hypertension, age ≥75 years, diabetes, prior stroke, vascular disease, age 65–74 years, and sex).

VAP-1, vascular adhesion protein-1; AF, atrial fibrillation; PAF, paroxysmal atrial fibrillation; PeAF, persistent atrial fibrillation; BMI, Body Mass Index; HF, heart failure; IS, Ischemic stroke; CHD, coronary heart disease; HBP, high blood pressure; DM, diabetes mellitus; COPD, chronic obstructive pulmonary disease; SBP, systolic blood pressure; DBP, diastolic blood pressure; HR, heart rate; WBC, white blood cell; Hb, hemoglobin; PLT, platelet; CRP, C-reactive protein; Scr, serum creatinine; Bun, blood urea nitrogen; UA, serum uric acid; eGFR, estimated glomerular filtrationrate; TG, triglyceride; TC, total cholesterol; HDL, high-density lipoprotein cholesterol; VLDL, very low density lipoprotein cholesterol; LDL, low density lipoprotein cholesterol; FBG, fasting blood-glucose; HbA1c, Glycated hemoglobin A1c; ALT, serum glutamic pyruvic transaminase; AST, serum glutamic oxaloacetic transaminase; TBil, total bilirubin; IBil, indirect bilirubin; ALB, serum albumin; CK, creatine kinase; CTnT, cardiac troponin T; NT-proBNP, N terminal brain natriuretic peptide precursor; LVEF, left ventricular ejection fraction; LAD, left atrial diameter; LVDD, left ventricular end-diastolic dimension; MACE, major adverse cardiovascular events.

### 3.2 Serum VAP-1 level and risk of AF

To explore the risk factors for the onset or progression of AF, we divided all 356 participants into two groups according to the presence or absence of AF (Supplementary Table S2). Univariate regression analyses indicated a positive correlation between VAP-1 level and the incidence of AF (*P* < 0.001; Supplementary Table S3). Moreover, old age, history of smoking or drinking, comorbidity with HF or coronary heart disease are all associated with an increased risk of AF. There were also statistically significant differences between groups in LAD, LVEF, NT-proBNP, some indicators of liver and kidney function and lipid profiles (Supplementary Table S3). To further clarify the relationship between VAP-1 levels and the risk of AF, multivariate regression analyses were conducted. After adjusting for the aforementioned potential confounding factors, elevated VAP-1 levels were still found to be associated with an increased risk of AF (*P* = 0.032, HR = 1.001, 95% CI = 1–1.002; Supplementary Table S4). The correlation of VAP-1 levels and AF risk was further validated through curve fitting (Supplementary Figure S1).

### 3.3 The correlation between VAP-1 and MACE

**Figure 2:**
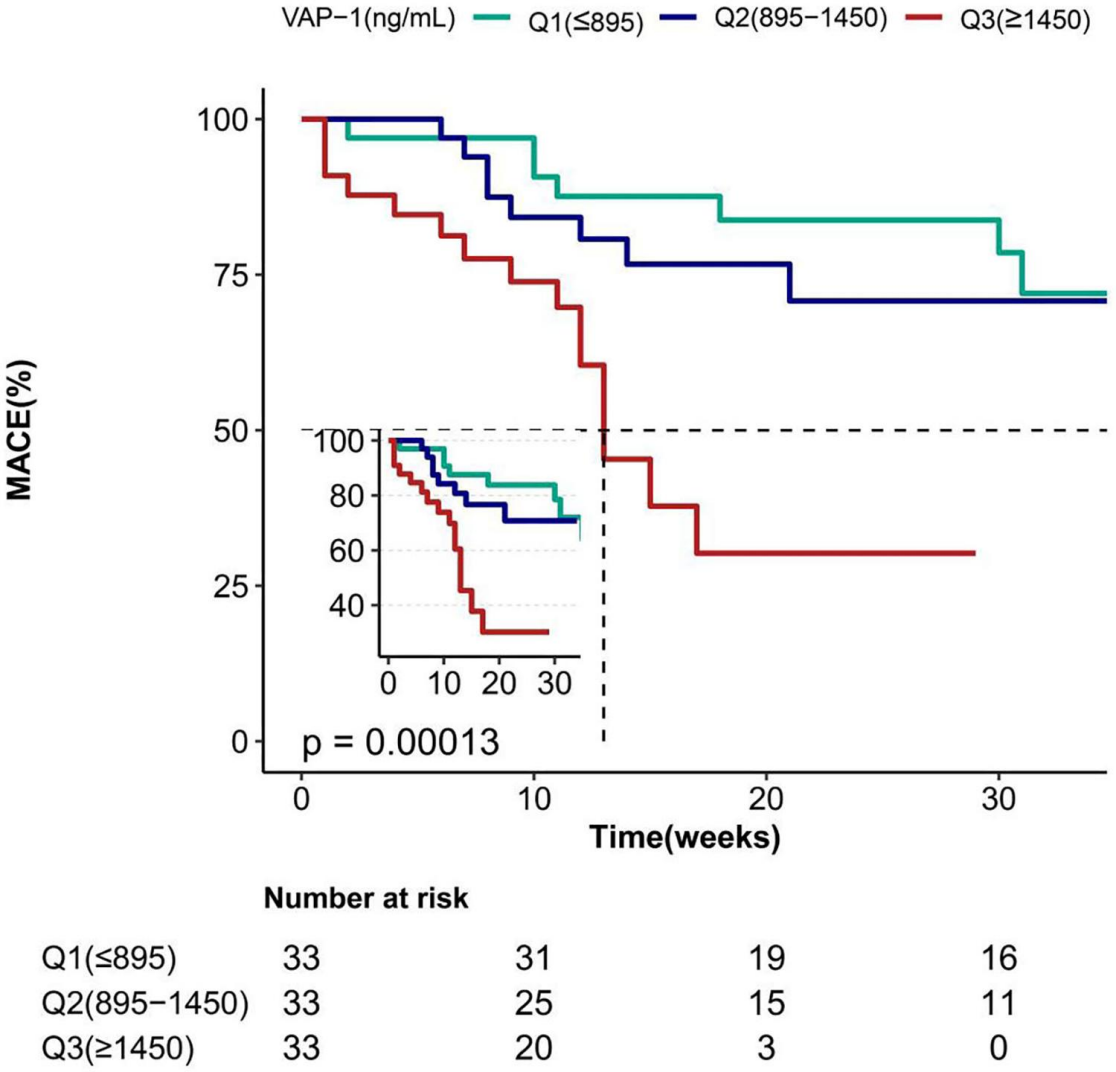
Kaplan–Meier survival curves for MACE of patients with AF. VAP-1, vascular adhesion protein-1; MACE, major adverse cardiovascular events; AF, atrial fibrillation.

**Figure 3:**
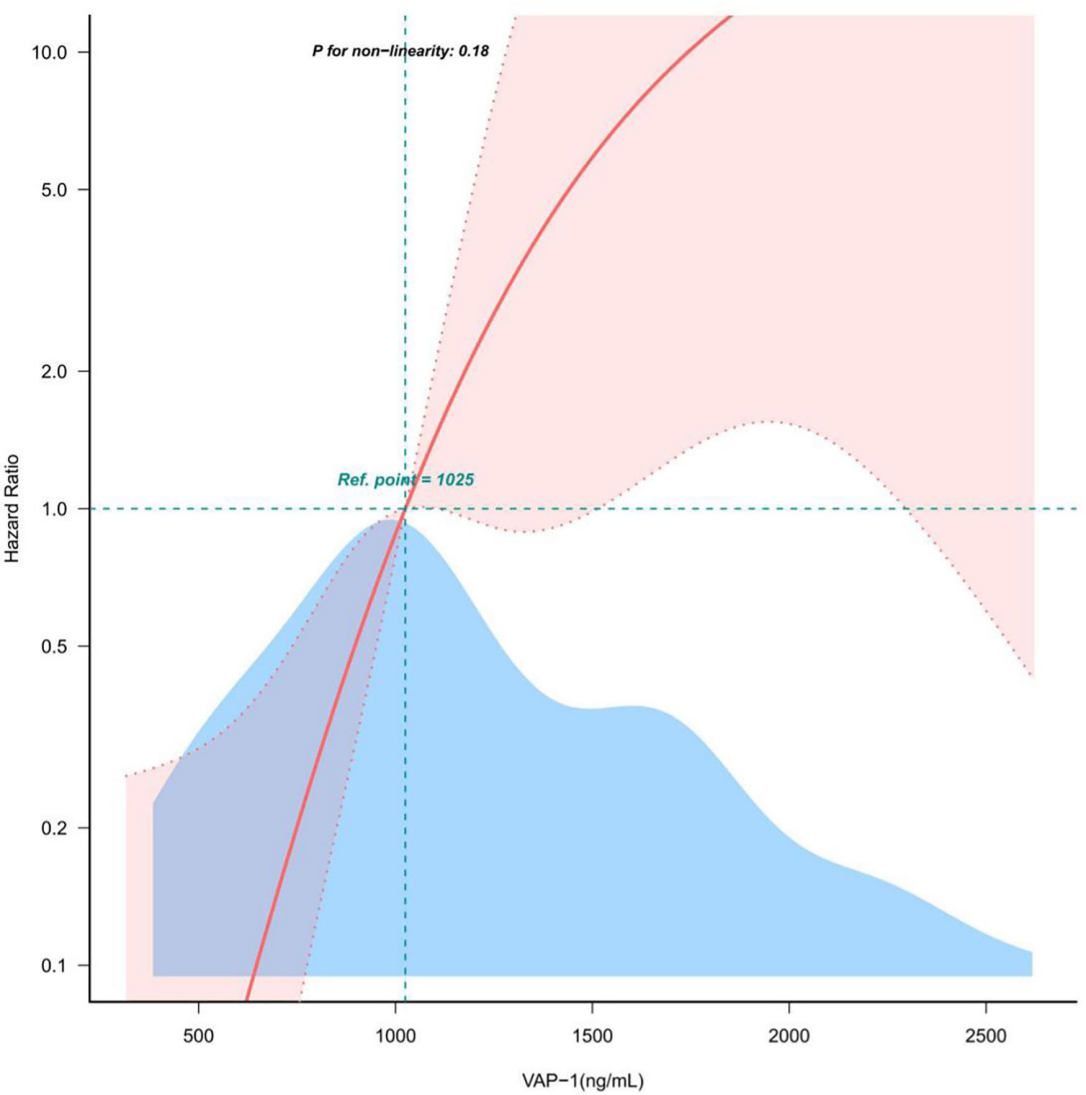
Relationship between VAP-1 and MACE in patients with AF. djusted for Gender, Age, HF, DBP, Scr, UA, eGFR, IBIL, ALB, NT-proBNP, D dimer, LAD, LVDD, AF types, IS, CHD, HBP, DM, COPD, Smoke, Drink, CHA2DS2-VASc, SBP, DBP, HR, WBC, Neutrophil, Lymphocyte, Hb, PLT, Scr, Bun, UA, eGFR, TG, TC, LDL, FBG, HbA1c, ALT, AST, IBIL, ALB, Sodium, Potassium, Chlorine, CK, CTnT, NT-proBNP, D dimer, LAD, LVDD. VAP-1, vascular adhesion protein-1; AF, atrial fibrillation; BMI, body mass index; HF, heart failure; DBP, diastolic blood pressure; Scr, serum creatinine; UA, serum uric acid; eGFR, estimated glomerular filtrationrate; IBil, indirect bilirubin; ALB, serum albumin; NT-proBNP, N terminal brain natriuretic peptide precursor; LAD, left atrial diameter; LVDD, left ventricular end-diastolic dimension; IS, Ischemic stroke; CHD, coronary heart disease; HBP, high blood pressure; DM, diabetes mellitus; COPD, chronic obstructive pulmonary disease; SBP, systolic blood pressure; HR, heart rate; WBC, white blood cell; Hb, hemoglobin; PLT, platelet; CRP, C-reactive protein; Bun, blood urea nitrogen; TG, triglyceride; TC, total cholesterol; HDL, high-density lipoprotein cholesterol; VLDL, very low density lipoprotein cholesterol; LDL, low density lipoprotein cholesterol; FBG, fasting blood-glucose; HbA1c, Glycated hemoglobin A1c; ALT, serum glutamic pyruvic transaminase; AST, serum glutamic oxaloacetic transaminase; TBil, total bilirubin; CK, creatine kinase; CTnT, cardiac troponin T; LVEF, left ventricular ejection fraction.

Clinical, demographic, biochemical, and radiographic data for patients with AF were analyzed using both univariate and multivariate methods to identify the predictors of MACE (Tables 2, 3). Univariate regression analysis showed that the occurrence of MACE in patients with AF was strongly associated with VAP-1 (*P* < 0.001), as well as age, HF, CHA2DS2-VASc, DBP, Hb, CRP, Scr, UA, eGFR, IBIL, ALB, NT-proBNP, D dimer, LAD, LVDD (Table 2). Meanwhile, further statistical methods were applied to explore and confirm the relationship of MACE and VAP-1 in AF patients. As shown in Table 3, in multiple regression analyses, VAP-1 levels were shown to be significantly associated with the occurrence of MACE in AF patients in both unadjusted (*P* < 0.001) model and adjusted models (model I: *P* < 0.001, model II: *P* = 0.028, model III: *P* = 0.021). Furthermore, the results remained consistent when the data were analyzed after stratification according to VAP-1 levels [(Q2 vs. Q1: HR = 5.28, 95% CI = 0.64–43.66), (Q3 vs. Q1: HR = 28.35, 95% CI = 2.82–284.92)], and notably, the test for trend was found to be statistically significant (*P* = 0.004). In line with these observations, Kaplan–Meier survival analysis revealed that the incidence of MACE events was higher among patients with elevated VAP-1 levels, with a maximum follow-up duration of 35 weeks (Q3 > Q2 > Q1, *P* = 0.00013, Figure 2). Consistently, the curve-fitting results indicated that the incidence of MACE in AF patients was positively correlated with VAP-1 levels (Figure 3). In the overall population, we were unable to identify a significant association between VAP-1 levels and the incidence of MACE (Supplementary Tables S5–S7).

**Table 2:** Univariate Cox regression analysis of risk factors associated with MACE in AF patients.

Variable	HR (95% CI)	*P* value
VAP-1 (ng/mL)	1.001 (1.0005, 1.0015)	<0.001
AF types (PAF)	1.4 (0.71, 2.77)	0.327
Gender (male)	0.78 (0.41, 1.5)	0.46
Age (years)	1.05 (1.01, 1.09)	0.008
BMI (Kg/㎡)	0.91 (0.84, 1)	0.051
HF (no)	6.15 (1.88, 20.08)	0.003
IS (no)	1.16 (0.51, 2.65)	0.727
CHD (no)	0.61 (0.29, 1.26)	0.18
HBP (no)	1.05 (0.52, 2.12)	0.892
DM (no)	1.5 (0.77, 2.95)	0.235
COPD (no)	0 (0, Inf)	0.997
Smoke (no)	0.51 (0.24, 1.09)	0.081
Drink (no)	0.19 (0.03, 1.36)	0.098
CHA2DS2-VASc, (score)	1.24 (1.04, 1.49)	0.018
SBP (mmHg)	0.99 (0.97, 1)	0.131
DBP (mmHg)	0.97 (0.94, 1)	0.039
HR (beats per min)	1.0031 (0.9809, 1.0259)	0.784
WBC (×10^9^/L)	0.87 (0.76, 1)	0.058
Neutrophil (×10^9^/L)	0.88 (0.75, 1.02)	0.096
Lymphocyte (×10^9^/L)	0.96 (0.52, 1.79)	0.902
Monocyte (×10^9^/L)	0.73 (0.09, 5.69)	0.768
Hb (g/L)	0.98 (0.96, 0.99)	0.004
PLT (×1,012/L)	0.9968 (0.9914, 1.0023)	0.252
CRP (mg/L)	1.2 (1.07, 1.34)	0.001
Scr (umol/L)	1.01 (1, 1.02)	0.04
Bun (mmol/L)	1.13 (0.99, 1.29)	0.071
UA (umol/L)	1.0025 (1.0002, 1.0048)	0.031
eGFR (ml/min)	0.97 (0.96, 0.99)	<0.001
TG (mmol/L)	0.9903 (0.5326, 1.8414)	0.975
TC (mmol/L)	0.81 (0.57, 1.16)	0.249
HDL (mmol/L)	0.37 (0.09, 1.47)	0.158
VLDL (mmol/L)	1.41 (0.59, 3.37)	0.434
LDL (mmol/L)	0.87 (0.59, 1.3)	0.495
FBG (mmol/L)	1.09 (0.89, 1.33)	0.395
HbA1c (%)	1.37 (0.89, 2.1)	0.148
ALT (U/L)	1.0053 (0.9891, 1.0219)	0.522
AST (U/L)	0.9943 (0.9848, 1.0038)	0.235
TBIL (mmol/L)	1.01 (0.97, 1.06)	0.505
IBIL (mmol/L)	1.12 (1.01, 1.23)	0.024
ALB (g/L)	0.88 (0.82, 0.95)	0.002
Sodium (mmol/L)	0.94 (0.86, 1.02)	0.118
Potassium (mmol/L)	1.11 (0.54, 2.26)	0.782
Chlorine (mmol/L)	0.98 (0.9, 1.06)	0.627
CK (U/L)	0.9982 (0.9955, 1.0008)	0.18
CTnT (pg/mL)	0.9984 (0.9964, 1.0005)	0.138
NT-proBNP (pg/mL)	1.0001 (1, 1.0001)	<0.001
D dimer (ug/mL)	1.21 (1.05, 1.38)	0.007
LVEF (%)	0.97 (0.95, 0.99)	0.004
LAD (mm)	1.04 (1.01, 1.08)	0.021
LVDD (mm)	1.05 (1.01, 1.09)	0.021

CHA2DS2-VASc is a scoring system for stroke risk assessment in patients with atrial fibrillation (congestive heart failure, hypertension, age ≥75 years, diabetes, prior stroke, vascular disease, age 65–74 years, and sex).

VAP-1, vascular adhesion protein-1; AF, atrial fibrillation; PAF, paroxysmal atrial fibrillation; BMI, Body Mass Index; HF, heart failure; IS, Ischemic stroke; CHD, coronary heart disease; HBP, high blood pressure; DM, diabetes mellitus; COPD, chronic obstructive pulmonary disease; SBP, systolic blood pressure; DBP, diastolic blood pressure; HR, heart rate; WBC, white blood cell; Hb, hemoglobin; PLT, platelet; CRP, C-reactive protein; Scr, serum creatinine; Bun, blood urea nitrogen; UA, serum uric acid; eGFR, estimated glomerular filtrationrate; TG, triglyceride; TC, total cholesterol; HDL, high-density lipoprotein cholesterol; VLDL, very low density lipoprotein cholesterol; LDL, low density lipoprotein cholesterol; FBG, fasting blood-glucose; HbA1c, Glycated hemoglobin A1c; ALT, serum glutamic pyruvic transaminase; AST, serum glutamic oxaloacetic transaminase; TBil, total bilirubin; IBil, indirect bilirubin; ALB, serum albumin; CK, creatine kinase; CTnT, cardiac troponin T; NT-proBNP, N terminal brain natriuretic peptide precursor; LVEF, left ventricular ejection fraction; LAD, left atrial diameter; LVDD, left ventricular end-diastolic dimension; MACE, major adverse cardiovascular events.

**Table 3:** Multivariate Cox regression analysis of risk factors associated with MACE in AF patients.

Variable	Categorization of VAP-1 levels	Non-adjusted model	*P* value	model I	*P* value	model II	*P* value	model III	*P* value
		HR (95% CI)		HR (95% CI)		HR (95% CI)		HR (95% CI)	
VAP-1(ng/mL)		1.001 (1–1.001)	<0.001	1.001 (1.001–1.002)	<0.001	1 (1–1)	0.028	1 (1–1)	0.021
VAP-1 (ng/mL)	Q1 (≤895)	1 (Ref)		1 (Ref)		1 (Ref)		1 (Ref)	
	Q2 (895–1,450)	1.84 (0.71–4.76)	0.212	1.64 (0.62–4.32)	0.319	2.3 (0.75–7.08)	0.146	5.28 (0.64–43.66)	0.123
	Q3 (≥1,450)	6.09 (2.34–15.9)	<0.001	6.06 (2.32–15.84)	<0.001	4.33 (1.29–14.53)	0.018	28.35 (2.82–284.92)	0.005
Trend test		2.55 (1.56–4.17)	<0.001	2.57 (1.56–4.23)	<0.001	2.05 (1.14–3.71)	0.017	5.33 (1.71–16.64)	0.004

Data presented are HR and 95% CIs.

CHA2DS2-VASc is a scoring system for stroke risk assessment in patients with atrial fibrillation (congestive heart failure, hypertension, age ≥ 75 years, diabetes, prior stroke, vascular disease, age 65–74 years, and sex).

Non-adjusted Model: We did not adjust any covariants.

Model I: We adjusted for Gender, Age.

Model II: We adjusted for Model I + HF, DBP, Scr, UA, eGFR, IBIL, ALB, NT-proBNP, D dimer, LAD, LVDD.

Model III: We adjusted for Model II + AF types, IS, CHD, HBP, DM, COPD, Smoke, Drink, CHA2DS2-VASc, SBP, DBP, HR, WBC, Neutrophil, Lymphocyte, Hb, PLT, Scr, Bun, UA, eGFR, TG, TC, LDL, FBG, HbA1c, ALT, AST, IBIL, ALB, Sodium, Potassium, Chlorine, CK, CTnT, NT-proBNP, D dimer, LAD, LVDD.

In each case, the model is not adjusted for the variable itself.

VAP-1, vascular adhesion protein-1; AF, atrial fibrillation; BMI, Body Mass Index; HF, heart failure; DBP, diastolic blood pressure; Scr, serum creatinine; UA, serum uric acid; eGFR, estimated glomerular filtrationrate; IBil, indirect bilirubin; ALB, serum albumin; NT-proBNP, N terminal brain natriuretic peptide precursor; LAD, left atrial diameter; LVDD, left ventricular end-diastolic dimension; IS, Ischemic stroke; CHD, coronary heart disease; HBP, high blood pressure; DM, diabetes mellitus; COPD, chronic obstructive pulmonary disease; SBP=systolic blood pressure; HR, heart rate; WBC, white blood cell; Hb, hemoglobin; PLT, platelet; CRP, C-reactive protein; Bun, blood urea nitrogen; TG, triglyceride; TC, total cholesterol; HDL, high-density lipoprotein cholesterol; VLDL, very low density lipoprotein cholesterol; LDL, low density lipoprotein cholesterol; FBG, fasting blood-glucose; HbA1c, Glycated hemoglobin A1c; ALT, serum glutamic pyruvic transaminase; AST, serum glutamic oxaloacetic transaminase; TBil, total bilirubin; CK, creatine kinase; CTnT, cardiac troponin T; LVEF, left ventricular ejection fraction.

## 4 Discussion

In this retrospective cohort study, we observed a positive correlation between high plasma VAP-1 levels and the risk of AF. Notably, our study revealed a robust correlation between elevated soluble VAP-1 levels and the occurrence of MACE in AF patients, which persisted even after accounting for other confounding variables. To the best of our knowledge, this is the first clinical study that specifically investigate the relationship between VAP-1 and AF as well as its prognosis, which may hold potential clinical significance for the management of AF.

Our findings indicate that elevated plasma VAP-1 levels are significantly associated with an increased risk of AF, which may hint at a potential role for VAP-1 in the pathogenesis of atrial fibrillation. The pathogenesis of AF is highly complex and multifactorial. A considerable amount of research has demonstrated that AF patients exhibit heightened inflammatory responses in the heart or systemic circulation compared to individuals with sinus rhythm (24). This is characterized by elevated levels of inflammatory cells, such as neutrophils and macrophages, as well as various inflammatory mediators, including proinflammatory cytokines, C-reactive protein (CRP), heat shock proteins (HSPs), transforming growth factor-beta (TGF-β), platelet-derived growth factor (PDGF), and myeloperoxidase (MPO), NOD-like receptor family pyrin domain containing 3 (NLRP3) inflammasome (25). These factors can collectively contribute to the electrophysiological, structural, and autonomic remodeling of the atria, thereby promoting the initiation and progression of AF. The role of reactive oxygen species (ROS) in atrial fibrillation (AF) has also garnered significant attention, with evidence suggesting that ROS contribute to the onset and perpetuation of AF by inflicting oxidative damage on critical biomolecules, namely proteins, lipids, and DNA (26). In addition to causing direct cellular injury, ROS have the capacity to exacerbate inflammation and promote fibrosis by triggering various inflammatory signaling pathways such as nuclear factor kappa B (NF-κB), transforming growth factor beta (TGF-β), and the Nod-like receptor protein 3 (NLRP3) inflammasome, thereby driving AF progression (27). VAP-1, as an adhesion molecule, has been demonstrated to facilitate the infiltration of immune cells in the myocardium by regulating leukocyte migration across vascular endothelial cells and tissues. Moreover, VAP-1 serves as an enzyme that catalyzes the generation of H_2_O_2_ from methylamine and amino-propanone, directly elevating ROS levels (3). As such, we speculate that VAP-1 could induce cardiac remodeling and injury by augmenting myocardial inflammation and oxidative stress, thereby facilitating the development of adverse cardiovascular events. However, as VAP-1 was measured at the time of or after the diagnosis of AF, the temporal relationship remains unclear. The elevations in VAP-1 may be a consequence rather than a cause of AF, or the elevation of VAP-1 levels and the occurrence of AF may both be consequences of shared inflammatory or hemodynamic pathways. Overall, this speculation requires further experimental verification.

Multiple biomarkers have been identified to predict the genesis and perpetuation of AF. These biological markers are closed related to the pathophysiological mechanisms and risk factors of AF such as atrial stress, inflammation, endothelial dysfunction, myocardial fibrosis, kidney dysfunction, coronary artery calcium, mitral annular calcium, electrocardiographic P-wave, etc. (28). It is now clear that systemic and cardiac inflammation contribute to the development of AF. Several pro-inflammatory biomarkers, including CRP, Interleukin-1, and tumor necrosis factor, are implicated in the initiation and progression of AF. Among them, CRP has been extensively studied and were considered to be associated with increased risk of AF, however, its predictive value for AF development and prognosis were not satisfactory (29). Therefore, the identification of highly sensitive and specific biomarkers for atrial fibrillation (AF) is of great clinical significance. In our study, VAP-1 was demonstrated to be independently associated with the risk of AF. Serving as an inflammatory mediator, VAP-1 functions both as an adhesion molecule and an amine oxidase. It appears to be more than a nonspecific marker of systemic inflammation and may actively participate in the pathogenesis of AF. Indeed, VAP-1 has been reported to be associated with progressive cardiac remodeling or injury (30–32), which is linked to the pathophysiology of AF. Moreover, prior researches has demonstrated the predictive value of plasma VAP-1 for adverse cardiovascular events in several different diseases and populations, including patients with coronary artery disease, type 2 diabetes mellitus, those undergoing hemodialysis, and individuals aged ≥50 years without a prior history of major adverse cardiovascular events (MACE) (8, 20–22). In the present study, we found that elevated plasma VAP-1 levels were independently associated with MACE in patients with AF, the occurrence of MACE events increased with increasing VAP-1 levels after a maximum follow-up of 35 weeks. Therefore, VAP-1 may have the potential to predict the incidence and outcomes in patients with AF. However, further research is needed to verify the predictive value of VAP-1 and to define the appropriate cut-off levels of VAP-1 in the context of AF. And its potential use as a therapeutic target for AF warrants further investigation in preclinical and clinical study.

In summary, our research provides initial evidence that elevated VAP-1 levels are associated with higher AF prevalence and increased MACE risk in AF patients. Despite strict criteria and adjustment for confounders, limitations remain. First, the study population was from a single hospital, potentially limiting generalizability, and there is inevitable selection bias due to its retrospective nature. Moreover, although we collected detailed patient characteristics, including comorbidities and biochemical indicators, and adjusted for potential confounding factors, there may still be unmeasured confounders due to variables not included in our dataset, such as patients' treatment and medication data (e.g., anticoagulation, heart failure therapy, anti-inflammatory drugs). Given the diversity and complexity of the aforementioned covariates, we will continue to increase the sample size in future studies to meet the requirements of statistical analysis and to validate and ensure the reliability of the results. Furthermore, the sample size was relatively small (*n* = 336), highlighting the need for larger studies. Lastly, all-cause mortality was not included as an endpoint due to COVID-19.

## 5 Conclusion

Our results suggest that elevated VAP-1 levels might substantially augment the prevalence of AF and the incidence of MACE among AF patients. VAP-1 could potentially serve as a valuable biomarker for predicting the onset and prognosis of AF.

## Data Availability

The raw data supporting the conclusions of this article will be made available by the authors, without undue reservation.
