# Supplementary material for: Correlation between vascular adhesion protein-1 and major adverse cardiovascular events in patients with atrial fibrillation
Source: Front Cardiovasc Med. 2025 Nov 21;12:1684589. doi: 10.3389/fcvm.2025.1684589 (PMC12678388; doi:10.3389/fcvm.2025.1684589)
Supplement: Supplementary file 1 [file Datasheet1.pdf]

Supplementary Material

1 This file includes:

Supplementary Figure

Supplementary Figure 1. Correlation of VAP-1 level with AF incidence

Supplementary Table 1-7

Supplementary Table 1. Comparative analysis before and after imputation of missing data

Supplementary Table 2. Baseline characteristics of patients grouped according to presence or absence of AF in all patients

Supplementary Table 3. Univariate analysis of association between VAP-1 and AF

Supplementary Table 4. Multivariate analysis of association between VAP-1 and AF

Supplementary Table 5. Baseline characteristics of all participants grouped according to VAP-1 level

Supplementary Table 6. Univariate Cox regression analysis of risk factors associated with MACE in all patients

Supplementary Table 7. Multivariate Cox regression analysis of risk factors associated with MACE in all patients

2 Supplementary Figure

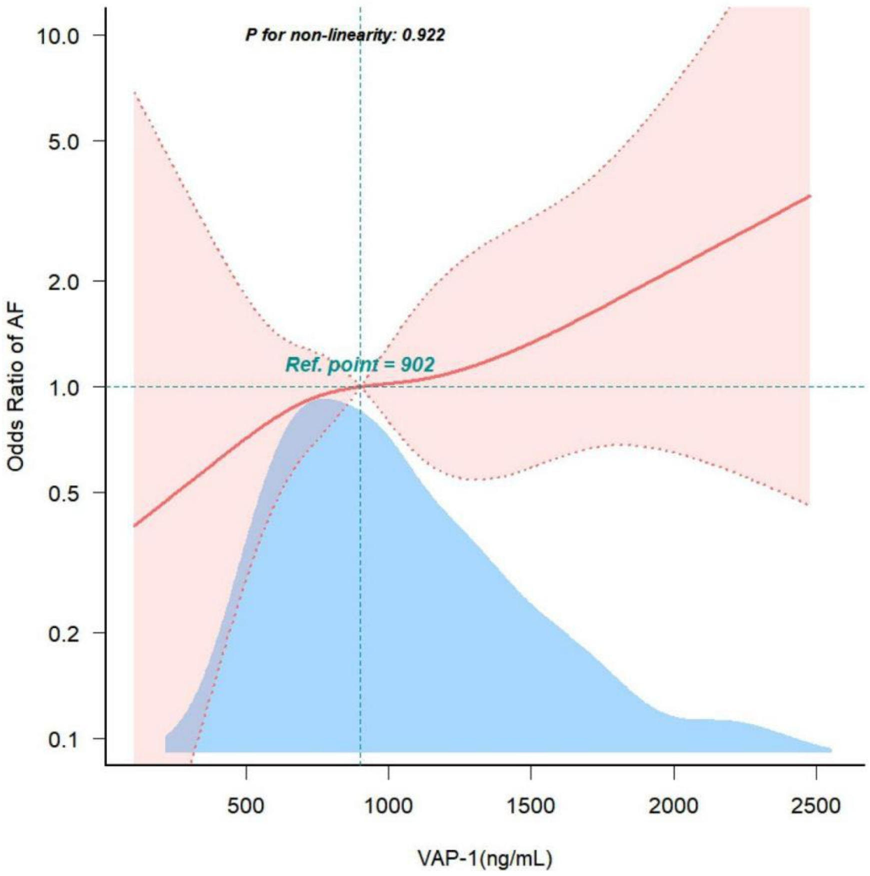

Supplementary Fig 1. Correlation of VAP-1 level with AF incidence

Adjusted for Gender, Age, HF, CHD, HBP, DM, Smoke, Drink, HR, WBC, Hb, PLT, Bun, UA, eGFR, TG, LDL, FBG, HbA1c, AST, TBIL, IBIL, CTnT, LVEF, LAD, LVDD, CHA2DS2-VASc, BMI, SBP, DBP, Neutrophil, Lymphocyte, CRP, TC, VLDL, ALT, Sodium, Potassium, Chlorine.

Notes: CHA2DS2-VASc is a scoring system for stroke risk assessment in patients with atrial fibrillation (congestive heart failure, hypertension, age ≥ 75 years, diabetes, prior stroke, vascular disease, age 65–74 years, and sex).

Abbreviations: AF = atrial fibrillation; VAP-1 =Vascular adhesion protein-1; HF = heart failure; CHD = coronary heart disease; HBP = high blood pressure; DM= diabetes mellitus; HR = heart rate; WBC = white blood cell; Hb = hemoglobin; PLT = platelet; Bun = blood urea nitrogen; UA = serum uric acid; eGFR = estimated glomerular filtrationrate; TG = triglyceride; LDL = low density lipoprotein cholesterol; FBG = fasting blood-glucose; HbA1c = Glycated hemoglobin A1c; AST = serum glutamic oxaloacetic transaminase; TBil = total bilirubin; IBil = indirect bilirubin; CTnT = cardiac troponin T; LVEF = left ventricular ejection fraction; LAD = left atrial diameter; LVDD = left ventricular end-diastolic dimension; BMI = Body Mass Index; SBP = systolic blood pressure; DBP = diastolic blood pressure; CRP = C-reactive protein; TC = total cholesterol; VLDL = very low density lipoprotein cholesterol; ALT = serum glutamic pyruvic transaminase.

3 Supplementary Tables

Supplementary Table 1. Comparative analysis before and after imputation of missing data.

Variables	Total (n = 336)	imputation of missing data		P value
		before (n = 171)	after (n = 165)	
VAP-1(ng/mL)	1010.8 ± 502.4	1010.8 ± 502.8	1010.8 ± 502.8	1
Gender (male), n (%)	476 (70.8)	238 (70.8)	238 (70.8)	1
Age (years)	67.7 ± 11.9	67.7 ± 11.9	67.7 ± 11.9	1
BMI (Kg/㎡)	24.5 ± 3.5	24.5 ± 3.6	24.5 ± 3.6	1
AF, n (%)	198 (29.5)	99 (29.5)	99 (29.5)	1
HF, n (%)	330 (49.1)	165 (49.1)	165 (49.1)	1
IS, n (%)	86 (12.8)	43 (12.8)	43 (12.8)	1
CHD, n (%)	348 (51.8)	174 (51.8)	174 (51.8)	1
HBP, n (%)	450 (67.0)	225 (67)	225 (67)	1

DM, n (%)	194 (28.9)	97 (28.9)	97 (28.9)	1
COPD, n (%)	58 (8.6)	29 (8.6)	29 (8.6)	1
Smoke, n (%)	272 (40.5)	136 (40.5)	136 (40.5)	1
Drink, n (%)	126 (18.8)	63 (18.8)	63 (18.8)	1
CHA2DS2-VASc, (score)	3.5 ± 1.6	3.5 ± 1.6	3.5 ± 1.6	1
SBP (mmHg)	134.4 ± 21.2	134.4 ± 21.2	134.4 ± 21.2	1
DBP (mmHg)	78.0 ± 13.3	78.0 ± 13.3	78.0 ± 13.3	1
HR (beats per minute)	80.0 ± 14.3	80.0 ± 14.4	80.0 ± 14.2	1
WBC (×109/L)	7.1 ± 2.5	7.1 ± 2.5	7.1 ± 2.5	1
Neutrophil (×109/L)	5.2 ± 2.6	5.2 ± 2.6	5.2 ± 2.6	1
Lymphocyte (×109/L)	1.5 ± 0.6	1.5 ± 0.6	1.5 ± 0.6	1
Monocyte (×109/L)	0.5 ± 0.2	0.5 ± 0.2	0.5 ± 0.2	1
Hb (g/L)	132.9 ± 17.7	132.9 ± 17.8	132.9 ± 17.6	1

PLT (×1012/L)	196.7 ± 58.9	196.7 ± 59.5	196.7 ± 58.4	1
CRP (mg/L)	5.7 ± 2.1	5.7 ± 2.2	5.7 ± 2.1	1
Scr (umol/L)	82.8 ± 30.6	82.8 ± 30.8	82.8 ± 30.4	1
Bun (mmol/L)	6.4 ± 2.3	6.4 ± 2.4	6.4 ± 2.3	1
UA (umol/L)	378.9 ± 126.8	378.9 ± 127.4	378.9 ± 126.3	1
eGFR (ml/min)	86.5 ± 29.4	86.5 ± 29.5	86.5 ± 29.3	1
TG (mmol/L)	1.1 (0.8, 1.5)	1.1 (0.8, 1.5)	1.1 (0.8, 1.5)	0.9
TC (mmol/L)	3.9 ± 1.0	3.9 ± 1.0	3.9 ± 1.0	1
HDL (mmol/L)	1.1 ± 0.3	1.1 ± 0.3	1.1 ± 0.3	1
VLDL (mmol/L)	0.4 (0.3, 0.6)	0.4 (0.3, 0.6)	0.4 (0.3, 0.6)	0.922
LDL (mmol/L)	2.4 ± 0.9	2.4 ± 0.9	2.4 ± 0.9	1
FBG (mmol/L)	6.1 ± 2.0	6.1 ± 2.0	6.1 ± 1.9	1
HbA1c (%)	6.2 ± 0.9	6.2 ± 1.0	6.2 ± 0.9	1

ALT (U/L)	19.0 (13.0, 31.0)	19.0 (13.0, 31.5)	19.0 (13.0, 31.0)	0.926
AST (U/L)	22.0 (16.0, 34.0)	22.0 (16.0, 33.0)	22.0 (16.0, 35.0)	0.727
TBIL (mmol/L)	11.5 (8.7, 16.2)	11.5 (8.7, 16.2)	11.6 (8.7, 16.2)	0.962
IBIL (mmol/L)	5.0 (3.7, 6.7)	5.0 (3.7, 6.7)	5.0 (3.7, 6.7)	0.894
ALB (g/L)	38.9 ± 4.6	38.9 ± 4.6	38.9 ± 4.5	1
Sodium (mmol/L)	140.9 ± 3.7	140.9 ± 3.7	140.9 ± 3.7	1
Potassium (mmol/L)	3.9 ± 0.4	3.9 ± 0.4	3.9 ± 0.4	1
Chlorine (mmol/L)	104.2 ± 3.8	104.2 ± 3.8	104.2 ± 3.8	1
CK (U/L)	90.0 (60.0, 179.0)	89.5 (59.2, 177.0)	91.0 (60.0, 187.2)	0.785
CTnT (pg/mL)	17.5 (5.0, 89.0)	17.5 (5.0, 81.0)	17.5 (5.0, 91.8)	0.857
NT-proBNP (pg/mL)	657.0 (102.0, 2988.0)	647.0 (101.2, 2933.8)	689.5 (102.0, 3147.8)	0.832
D dimer (ug/mL)	0.4 (0.2, 0.9)	0.4 (0.2, 0.8)	0.4 (0.2, 0.9)	0.72
LVEF (%)	53.9 ± 14.8	53.9 ± 14.8	53.9 ± 14.8	1

LAD (mm)	45.5 ± 7.8	45.5 ± 7.8	45.5 ± 7.8	1
LVDD (mm)	52.5 ± 8.6	52.5 ± 8.6	52.5 ± 8.6	1
MACE, n (%)	166 (24.7)	83 (24.7)	83 (24.7)	1
Time of MACE (weeks)	20.0 (14.0, 32.0)	20.0 (14.0, 32.0)	20.0 (14.0, 32.0)	1

Notes: Percentage calculated from the total population; some factors total <100% due to missing data.

Abbreviations: HF = heart failure; VAP-1 =Vascular adhesion protein-1; IS = Ischemic stroke; CHD = coronary heart disease; non-CHD = patients without coronary heart disease; AP = angor pectoris; AMI = acute myocardial infarction; BMI = Body Mass Index; Af = atrial fibrillation; HBP = high blood pressure; DM= diabetes mellitus; COPD = chronic obstructive pulmonary disease; SBP = systolic blood pressure; DBP = diastolic blood pressure; HR = heart rate; WBC = white blood cell; Hb = hemoglobin; PLT = platelet; CRP = C-reactive protein; Scr = serum creatinine; Bun = blood urea nitrogen; UA = serum uric acid; eGFR = estimated glomerular filtrationrate; TG = triglyceride; TC = total cholesterol; HDL = high-density lipoprotein cholesterol; VLDL = very low density lipoprotein cholesterol; LDL = low density lipoprotein cholesterol; FBG = fasting blood-glucose; HbA1c = Glycated hemoglobin A1c; ALT = serum glutamic pyruvic transaminase; AST = serum glutamic oxaloacetic transaminase; TBil = total bilirubin; IBil = indirect bilirubin; ALB = serum albumin; CK = creatine kinase; CTnT = cardiac troponin T; NT-proBNP = N terminal brain natriuretic peptide precursor; LVEF = left ventricular ejection fraction; LAD = left atrial diameter; LVDD = left ventricular end-diastolic dimension; MACE = major adverse cardiovascular events.

Supplementary Table 2. Baseline characteristics of patients grouped according to presence or absence of atrial fibrillation in all patients.

Variables	Total (n = 336)	AF		P value
		no (n = 237)	yes (n = 99)	
VAP-1(ng/mL)	1010.8 ± 502.8	931.3 ± 433.9	1201.3 ± 599.1	< 0.001
Gender (male), n (%)	238 (70.8)	170 (71.7)	68 (68.7)	0.576
Age (years)	67.7 ± 11.9	65.8 ± 12.4	72.3 ± 9.3	< 0.001
BMI (Kg/㎡)	24.5 ± 3.6	24.6 ± 3.4	24.2 ± 3.8	0.334
HF, n (%)	165 (49.1)	89 (37.6)	76 (76.8)	< 0.001
IS, n (%)	43 (12.8)	28 (11.8)	15 (15.2)	0.404
CHD, n (%)	174 (51.8)	142 (59.9)	32 (32.3)	< 0.001
HBP, n (%)	225 (67.0)	158 (66.7)	67 (67.7)	0.858
DM, n (%)	97 (28.9)	71 (30)	26 (26.3)	0.496
COPD, n (%)	29 (8.6)	19 (8)	10 (10.1)	0.535

SMOKE, n (%)	136 (40.5)	107 (45.1)	29 (29.3)	0.007
drink, n (%)	63 (18.8)	52 (21.9)	11 (11.1)	0.02
CHA2DS2-VASc, (score)	3.5 ± 1.6	3.4 ± 1.5	3.8 ± 1.7	0.019
SBP (mmHg)	134.4 ± 21.2	134.9 ± 20.9	133.4 ± 21.8	0.557
DBP (mmHg)	78.0 ± 13.3	78.4 ± 13.0	76.9 ± 14.1	0.336
HR (beats per minute)	80.0 ± 14.4	78.5 ± 13.5	84.0 ± 16.0	0.002
WBC (×109/L)	7.1 ± 2.5	7.3 ± 2.5	6.7 ± 2.5	0.044
Neutrophil (×109/L)	5.2 ± 2.6	5.4 ± 2.7	4.8 ± 2.4	0.051
Lymphocyte (×109/L)	1.5 ± 0.6	1.5 ± 0.6	1.3 ± 0.5	0.002
Monocyte (×109/L)	0.5 ± 0.2	0.5 ± 0.2	0.5 ± 0.2	0.134
Hb (g/L)	132.9 ± 17.8	134.5 ± 16.9	129.3 ± 19.4	0.018
PLT (×1012/L)	196.7 ± 59.5	206.0 ± 56.3	175.0 ± 61.2	< 0.001
CRP (mg/L)	5.7 ± 2.2	5.7 ± 2.1	5.9 ± 2.4	0.426

Scr (umol/L)	82.8 ± 30.8	80.1 ± 29.8	89.4 ± 32.2	0.013
Bun (mmol/L)	6.4 ± 2.4	6.1 ± 2.3	7.0 ± 2.3	0.001
UA (umol/L)	378.9 ± 127.4	359.2 ± 113.0	425.6 ± 146.8	< 0.001
eGFR (ml/min)	86.5 ± 29.5	90.6 ± 28.7	76.8 ± 29.2	< 0.001
TG (mmol/L)	1.1 (0.8, 1.5)	1.2 (0.9, 1.7)	0.9 (0.7, 1.2)	< 0.001
TC (mmol/L)	3.9 ± 1.0	4.0 ± 1.0	3.5 ± 0.9	< 0.001
HDL (mmol/L)	1.1 ± 0.3	1.1 ± 0.3	1.1 ± 0.3	0.498
VLDL (mmol/L)	0.4 (0.3, 0.6)	0.4 (0.3, 0.6)	0.4 (0.2, 0.5)	0.115
LDL (mmol/L)	2.4 ± 0.9	2.5 ± 0.9	2.1 ± 0.9	< 0.001
FBG (mmol/L)	6.1 ± 2.0	6.1 ± 2.0	6.2 ± 2.0	0.96
HbA1c (%)	6.2 ± 1.0	6.2 ± 1.0	6.2 ± 0.8	0.604
ALT (U/L)	19.0 (13.0, 31.5)	20.0 (13.0, 32.0)	18.0 (12.0, 28.8)	0.164
AST (U/L)	22.0 (16.0, 33.0)	22.0 (16.0, 36.0)	20.0 (17.0, 29.0)	0.243

TBIL (mmol/L)	11.5 (8.7, 16.2)	10.9 (8.3, 15.9)	13.1 (9.8, 17.5)	0.002
IBIL (mmol/L)	5.0 (3.7, 6.7)	4.6 (3.5, 6.3)	6.1 (4.4, 7.3)	< 0.001
ALB (g/L)	38.9 ± 4.6	39.4 ± 4.6	37.7 ± 4.4	0.001
Sodium (mmol/L)	140.9 ± 3.7	140.9 ± 3.6	140.7 ± 4.1	0.549
Potassium (mmol/L)	3.9 ± 0.4	3.9 ± 0.4	3.9 ± 0.5	0.08
Chlorine (mmol/L)	104.2 ± 3.8	104.4 ± 3.4	103.7 ± 4.5	0.129
CK (U/L)	89.5 (59.2, 177.0)	97.0 (63.0, 237.0)	79.0 (53.0, 126.0)	< 0.001
CTnT (pg/mL)	17.5 (5.0, 81.0)	15.5 (5.0, 175.2)	20.0 (7.0, 43.0)	0.933
NT-proBNP (pg/mL)	647.0 (101.2, 2933.8)	209.5 (67.5, 1650.0)	2383.0 (911.2, 6780.5)	< 0.001
D dimer (ug/mL)	0.4 (0.2, 0.8)	0.3 (0.2, 0.6)	0.5 (0.2, 1.3)	< 0.001
LVEF (%)	53.9 ± 14.8	55.6 ± 14.3	49.8 ± 15.2	0.001
LAD (mm)	45.5 ± 7.8	43.2 ± 6.3	50.8 ± 8.5	< 0.001
LVDD (mm)	52.5 ± 8.6	52.1 ± 8.5	53.6 ± 8.9	0.136

MACE, n (%)	83 (24.7)	44 (18.6)	39 (39.4)	< 0.001
Time of MACE (weeks)	20.0 (14.0, 32.0)	22.0 (17.0, 33.0)	15.0 (10.0, 30.0)	< 0.001

Notes: Percentage calculated from the total population; some factors total <100% due to missing data.

CHA2DS2-VASc is a scoring system for stroke risk assessment in patients with atrial fibrillation (congestive heart failure, hypertension, age ≥ 75 years, diabetes, prior stroke, vascular disease, age 65–74 years, and sex).

Abbreviations: AF = atrial fibrillation; VAP-1 =Vascular adhesion protein-1; IS = Ischemic stroke; CHD = coronary heart disease; non-CHD = patients without coronary heart disease; AP = angor pectoris; AMI = acute myocardial infarction; BMI = Body Mass Index; HF = heart failure; HBP = high blood pressure; DM= diabetes mellitus; COPD = chronic obstructive pulmonary disease; SBP = systolic blood pressure; DBP = diastolic blood pressure; HR = heart rate; WBC = white blood cell; Hb = hemoglobin; PLT = platelet; CRP = C-reactive protein; Scr = serum creatinine; Bun = blood urea nitrogen; UA = serum uric acid; eGFR = estimated glomerular filtrationrate; TG = triglyceride; TC = total cholesterol; HDL = high-density lipoprotein cholesterol; VLDL = very low density lipoprotein cholesterol; LDL = low density lipoprotein cholesterol; FBG = fasting blood-glucose; HbA1c = Glycated hemoglobin A1c; ALT = serum glutamic pyruvic transaminase; AST = serum glutamic oxaloacetic transaminase; TBil = total bilirubin; IBil = indirect bilirubin; ALB = serum albumin; CK = creatine kinase; CTnT = cardiac troponin T; NT-proBNP = N terminal brain natriuretic peptide precursor; LVEF = left ventricular ejection fraction; LAD = left atrial diameter; LVDD = left ventricular end-diastolic dimension; MACE = major adverse cardiovascular events.

Supplementary Table 3. Univariate analysis of association between VAP-1 and AF.

**Variables**	**OR (95%CI)**	**P value**
VAP-1 (ng/mL)	1 (1~1)	<0.001
Gender (male)	0.86 (0.52~1.44)	0.576
Age (years)	1.05 (1.03~1.08)	<0.001
BMI (Kg/㎡)	0.97 (0.9~1.03)	0.333
HF (no)	5.49 (3.22~9.39)	<0.001
IS (no)	1.33 (0.68~2.62)	0.405
CHD (no)	0.32 (0.19~0.52)	<0.001
HBP (no)	1.05 (0.63~1.73)	0.858
DM (no)	0.83 (0.49~1.41)	0.496
COPD (no)	1.29 (0.58~2.88)	0.536
Smoke (no)	0.5 (0.3~0.83)	0.007
Drink (no)	0.44 (0.22~0.89)	0.023
CHA2DS2-VASc, (score)	1.19 (1.03~1.38)	0.021
SBP (mmHg)	1 (0.99~1.01)	0.556
DBP (mmHg)	0.99 (0.97~1.01)	0.336
HR (beats per minute)	1.03 (1.01~1.04)	0.003
WBC (×109/L)	0.9 (0.82~1)	0.044
Neutrophil (×109/L)	0.91 (0.82~1)	0.053
Lymphocyte (×109/L)	0.49 (0.31~0.77)	0.002

Monocyte (×109/L)	0.4 (0.12~1.33)	0.135
Hb (g/L)	0.98 (0.97~1)	0.019
PLT (×1012/L)	0.99 (0.99~0.99)	<0.001
CRP (mg/L)	1.04 (0.94~1.16)	0.424
Scr (umol/L)	1.01 (1~1.02)	0.016
Bun (mmol/L)	1.17 (1.06~1.29)	0.002
UA (umol/L)	1 (1~1.01)	<0.001
eGFR (ml/min)	0.98 (0.98~0.99)	<0.001
TG (mmol/L)	0.45 (0.28~0.71)	0.001
TC (mmol/L)	0.59 (0.46~0.77)	<0.001
HDL (mmol/L)	0.74 (0.31~1.76)	0.494
VLDL (mmol/L)	0.67 (0.27~1.63)	0.375
LDL (mmol/L)	0.63 (0.48~0.83)	0.001
FBG (mmol/L)	1 (0.89~1.13)	0.959
HbA1c (%)	0.93 (0.72~1.21)	0.598
ALT (U/L)	1 (0.99~1.01)	0.784
AST (U/L)	0.99 (0.99~1)	0.08
TBIL (mmol/L)	1.05 (1.01~1.08)	0.006
IBIL (mmol/L)	1.12 (1.04~1.21)	0.005
ALB (g/L)	0.92 (0.87~0.97)	0.001
Sodium (mmol/L)	0.98 (0.92~1.04)	0.548

Potassium (mmol/L)	1.62 (0.94~2.77)	0.082
Chlorine (mmol/L)	0.95 (0.9~1.01)	0.129
CK (U/L)	1 (1~1)	0.021
CTnT (pg/mL)	1 (1~1)	0.014
NT-proBNP (pg/mL)	1 (1~1)	<0.001
D dimer (ug/mL)	1.23 (1.04~1.45)	0.015
LVEF (%)	0.97 (0.96~0.99)	0.001
LAD (mm)	1.15 (1.11~1.2)	<0.001
LVDD (mm)	1.02 (0.99~1.05)	0.138

Notes: CHA2DS2-VASc is a scoring system for stroke risk assessment in patients with atrial fibrillation (congestive heart failure, hypertension, age ≥ 75 years, diabetes, prior stroke, vascular disease, age 65–74 years, and sex).

Abbreviations: AF = atrial fibrillation; VAP-1 =Vascular adhesion protein-1; IS = Ischemic stroke; CHD = coronary heart disease; non-CHD = patients without coronary heart disease; AP = angor pectoris; AMI = acute myocardial infarction; BMI = Body Mass Index; HF = heart failure; HBP = high blood pressure; DM= diabetes mellitus; COPD = chronic obstructive pulmonary disease; SBP = systolic blood pressure; DBP = diastolic blood pressure; HR = heart rate; WBC = white blood cell; Hb = hemoglobin; PLT = platelet; CRP = C-reactive protein; Scr = serum creatinine; Bun = blood urea nitrogen; UA = serum uric acid; eGFR = estimated glomerular filtrationrate; TG = triglyceride; TC = total cholesterol; HDL = high-density lipoprotein cholesterol; VLDL = very low density lipoprotein cholesterol; LDL = low density lipoprotein cholesterol; FBG = fasting blood-glucose; HbA1c = Glycated hemoglobin A1c; ALT = serum glutamic pyruvic transaminase; AST = serum glutamic oxaloacetic transaminase; TBil = total bilirubin; IBil = indirect bilirubin; ALB = serum albumin; CK = creatine kinase; CTnT = cardiac troponin T; NT-proBNP = N terminal brain natriuretic peptide precursor; LVEF = left ventricular ejection fraction; LAD = left atrial diameter; LVDD = left ventricular end-diastolic dimension.

Supplementary Table 4. Multivariate analysis of association between VAP-1 and AF.

Variable	Non-adjusted Model	P value	Model I	P value	Model II	P value	Model III	P value
	OR (95%CI)		OR (95%CI)		OR (95%CI)		OR (95%CI)	
**VAP-1 (ng/mL)**	1.001 (1.001~1.002)	<0.001	1.001 (1~1.001)	<0.001	1.001 (1~1.002)	0.021	1.001 (1~1.002)	0.032

Notes: data presented are OR and 95% CIs.

CHA2DS2-VASc is a scoring system for stroke risk assessment in patients with atrial fibrillation (congestive heart failure, hypertension, age ≥ 75 years, diabetes, prior stroke, vascular disease, age 65–74 years, and sex).

Non-adjusted Model: We did not adjust any covariants.

Model I: Adjusted for Gender, Age.

Model II: Adjusted for the variables in Model I plus HF, CHD, HBP, DM, Smoke, Drink, HR, WBC, Hb, PLT, Bun, UA, eGFR, TG, LDL, FBG, HbA1c, AST, TBIL, IBIL, CTnT, LVEF, LAD, LVDD.

Model III: Adjusted for the variables in Model II plus CHA2DS2-VASc, BMI, SBP, DBP, Neutrophil, Lymphocyte, CRP, TC, VLDL, ALT, Sodium, Potassium, Chlorine.

In each case, the model is not adjusted for the variable itself.

Abbreviations: AF = atrial fibrillation; VAP-1 =Vascular adhesion protein-1; HF = heart failure; CHD = coronary heart disease;HBP = high blood pressure; DM= diabetes mellitus; HR = heart rate; WBC = white blood cell; Hb = hemoglobin; PLT = platelet; Bun = blood urea nitrogen;

UA = serum uric acid; eGFR = estimated glomerular filtrationrate; TG = triglyceride; LDL = low density lipoprotein cholesterol; FBG = fasting blood-glucose; HbA1c = Glycated hemoglobin A1c; AST = serum glutamic oxaloacetic transaminase; TBil = total bilirubin; IBil = indirect bilirubin; CTnT = cardiac troponin T; LVEF = left ventricular ejection fraction; LAD = left atrial diameter; LVDD = left ventricular end-diastolic dimension; BMI = Body Mass Index; SBP = systolic blood pressure; DBP = diastolic blood pressure; CRP = C-reactive protein; TC = total cholesterol; VLDL = very low density lipoprotein cholesterol; ALT = serum glutamic pyruvic transaminase.

Supplementary Table 5. Baseline characteristics of all participants grouped according to VAP-1 level.

Variables	VAP-1(ng/mL)				P value
	**Total**	**Q1（≤729）**	**Q2（729-1130）**	**Q3 （≥1130）**	
	**(n = 336)**	**(n = 112)**	**(n = 112)**	**(n = 112)**	
AF, n (%)	99 (29.5)	21 (18.8)	33 (29.5)	45 (40.2)	0.002
Gender (male), n (%)	238 (70.8)	77 (68.8)	81 (72.3)	80 (71.4)	0.829
Age (years)	67.7 ± 11.9	61.6 ± 14.0	70.1 ± 10.1	71.5 ± 8.5	< 0.001
BMI (Kg/㎡)	24.5 ± 3.6	25.3 ± 3.5	24.1 ± 3.3	24.1 ± 3.7	0.01
HF, n (%)	165 (49.1)	39 (34.8)	50 (44.6)	76 (67.9)	< 0.001
IS, n (%)	43 (12.8)	11 (9.8)	18 (16.1)	14 (12.5)	0.373
CHD, n (%)	174 (51.8)	61 (54.5)	59 (52.7)	54 (48.2)	0.628
HBP, n (%)	225 (67.0)	86 (76.8)	69 (61.6)	70 (62.5)	0.025

DM, n (%)	97 (28.9)	24 (21.4)	33 (29.5)	40 (35.7)	0.061
COPD, n (%)	29 (8.6)	9 (8)	9 (8)	11 (9.8)	0.86
Smoke, n (%)	136 (40.5)	51 (45.5)	49 (43.8)	36 (32.1)	0.086
Drink, n (%)	63 (18.8)	27 (24.1)	21 (18.8)	15 (13.4)	0.121
CHA2DS2-VASc, (score)	3.5 ± 1.6	3.1 ± 1.6	3.5 ± 1.6	3.9 ± 1.5	0.002
SBP (mmHg)	134.4 ± 21.2	137.1 ± 20.9	130.3 ± 18.6	135.9 ± 23.4	0.035
DBP (mmHg)	78.0 ± 13.3	80.5 ± 13.4	76.3 ± 11.8	77.2 ± 14.4	0.043
HR (beats per minute)	80.0 ± 14.4	80.3 ± 14.9	78.9 ± 14.0	80.9 ± 14.4	0.575
WBC (×109/L)	7.1 ± 2.5	7.6 ± 2.8	6.8 ± 2.3	7.0 ± 2.4	0.059
Neutrophil(×109/L)	4.6 (3.3, 6.7)	4.8 (3.2, 7.7)	4.4 (3.3, 6.0)	4.4 (3.4, 6.4)	0.329
Lymphocyte (×109/L)	1.5 ± 0.6	1.6 ± 0.6	1.4 ± 0.6	1.4 ± 0.6	0.11
Monocyte (×109/L)	0.5 ± 0.2	0.5 ± 0.2	0.4 ± 0.2	0.5 ± 0.2	0.11

Hb (g/L)	132.9 ± 17.8	137.1 ± 17.8	131.3 ± 17.3	130.4 ± 17.8	0.011
PLT (×1012/L)	196.7 ± 59.5	217.6 ± 60.1	195.2 ± 55.2	178.0 ± 56.8	< 0.001
CRP (mg/L)	5.7 ± 2.2	5.6 ± 2.6	5.4 ± 1.1	6.2 ± 2.5	0.016
Scr (umol/L)	82.8 ± 30.8	75.7 ± 25.0	80.3 ± 29.0	92.8 ± 35.4	< 0.001
Bun (mmol/L)	6.4 ± 2.4	5.6 ± 2.0	6.3 ± 2.1	7.2 ± 2.7	< 0.001
UA (umol/L)	378.9 ± 127.4	374.7 ± 113.0	368.7 ± 119.0	393.7 ± 148.0	0.321
eGFR (ml/min)	86.5 ± 29.5	97.0 ± 28.8	88.7 ± 25.6	74.0 ± 29.4	< 0.001
TG (mmol/L)	1.1 (0.8, 1.5)	1.3 (1.0, 1.9)	1.1 (0.9, 1.5)	1.0 (0.8, 1.3)	< 0.001
TC (mmol/L)	3.9 ± 1.0	4.0 ± 1.0	4.0 ± 1.0	3.6 ± 1.0	0.002
HDL (mmol/L)	1.1 ± 0.3	1.1 ± 0.3	1.1 ± 0.3	1.1 ± 0.3	0.514
VLDL (mmol/L)	0.4 (0.3, 0.6)	0.4 (0.3, 0.6)	0.4 (0.3, 0.6)	0.4 (0.3, 0.6)	0.823
LDL (mmol/L)	2.4 ± 0.9	2.5 ± 1.0	2.6 ± 1.0	2.1 ± 0.8	< 0.001
FBG (mmol/L)	6.1 ± 2.0	6.0 ± 1.8	6.1 ± 1.7	6.4 ± 2.4	0.276

HbA1c (%)	6.2 ± 1.0	6.0 ± 0.9	6.1 ± 0.9	6.5 ± 1.0	0.003
ALT (U/L)	19.0 (13.0, 31.5)	21.0 (13.0, 35.5)	18.0 (13.0, 30.0)	21.0 (13.0, 30.0)	0.68
AST (U/L)	22.0 (16.0, 33.0)	21.0 (16.0, 33.2)	21.5 (17.0, 33.0)	23.0 (16.0, 33.0)	0.966
TBIL (mmol/L)	11.5 (8.7, 16.2)	11.4 (8.9, 15.2)	11.6 (8.4, 16.7)	12.8 (8.7, 18.8)	0.311
IBIL (mmol/L)	5.0 (3.7, 6.7)	4.9 (3.7, 6.2)	4.7 (3.6, 6.6)	5.3 (3.8, 7.4)	0.055
ALB (g/L)	38.9 ± 4.6	39.7 ± 4.6	38.4 ± 4.5	38.6 ± 4.5	0.066
Sodium (mmol/L)	140.9 ± 3.7	141.2 ± 3.6	141.2 ± 3.4	140.2 ± 4.1	0.066
Potassium (mmol/L)	3.9 ± 0.4	3.8 ± 0.4	3.9 ± 0.4	4.0 ± 0.5	0.024
Chlorine (mmol/L)	104.2 ± 3.8	104.2 ± 3.1	104.6 ± 3.6	103.8 ± 4.5	0.311
CK (U/L)	89.5 (59.2, 177.0)	96.0 (61.0, 215.5)	83.0 (61.0, 180.0)	90.5 (58.0, 138.5)	0.523
CTnT (pg/mL)	17.5 (5.0, 81.0)	10.0 (5.0, 126.0)	15.0 (5.0, 122.6)	20.0 (10.0, 52.8)	0.466
NT-proBNP (pg/mL)	647.0 (101.2, 2933.8)	197.0 (57.0, 1238.0)	480.0 (108.0, 2720.5)	2117.0 (339.0, 6044.0)	< 0.001
D dimer (ug/mL)	0.4 (0.2, 0.8)	0.3 (0.2, 0.6)	0.4 (0.2, 0.8)	0.5 (0.3, 1.1)	0.002

LVEF (%)	53.9 ± 14.8	59.8 ± 10.7	54.6 ± 15.3	47.3 ± 15.2	< 0.001
LAD (mm)	45.5 ± 7.8	42.9 ± 7.1	45.6 ± 7.9	47.9 ± 7.7	< 0.001
LVDD (mm)	52.5 ± 8.6	49.9 ± 5.9	52.6 ± 8.7	55.1 ± 9.9	< 0.001
MACE, n (%)	83 (24.7)	24 (21.4)	23 (20.5)	36 (32.1)	0.081
Time of MACE (weeks)	20.0 (14.0, 32.0)	30.0 (20.0, 34.2)	20.0 (16.0, 31.0)	15.0 (8.0, 22.8)	< 0.001

Notes: Percentage calculated from the total population; some factors total <100% due to missing data.

CHA2DS2-VASc is a scoring system for stroke risk assessment in patients with atrial fibrillation (congestive heart failure, hypertension, age ≥ 75 years, diabetes, prior stroke, vascular disease, age 65–74 years, and sex).

Abbreviations: VAP-1 =Vascular adhesion protein-1; AF = atrial fibrillation; BMI = Body Mass Index; HF = heart failure; IS = Ischemic stroke; CHD = coronary heart disease; HBP = high blood pressure; DM= diabetes mellitus; COPD = chronic obstructive pulmonary disease; SBP = systolic blood pressure; DBP = diastolic blood pressure; HR = heart rate; WBC = white blood cell; Hb = hemoglobin; PLT = platelet; CRP = C-reactive protein; Scr = serum creatinine; Bun = blood urea nitrogen; UA = serum uric acid; eGFR = estimated glomerular filtrationrate; TG = triglyceride; TC = total cholesterol; HDL = high-density lipoprotein cholesterol; VLDL = very low density lipoprotein cholesterol; LDL = low density lipoprotein cholesterol; FBG = fasting blood-glucose; HbA1c = Glycated hemoglobin A1c; ALT = serum glutamic pyruvic transaminase; AST = serum glutamic oxalacetic transaminase; TBil = total bilirubin; IBil = indirect bilirubin; ALB = serum albumin; CK = creatine kinase; CTnT = cardiac troponin T; NT-proBNP = N terminal brain natriuretic peptide precursor; LVEF = left ventricular ejection fraction; LAD = left atrial diameter; LVDD = left ventricular end-diastolic dimension; MACE = major adverse cardiovascular events.

Supplementary Table 6. Univariate Cox regression analysis of risk factors associated with MACE in all patients.

Variable	HR (95%CI)	P value
VAP-1 (ng/mL)	1.0011 (1.0007,1.0015)	< 0.001
AF (no)	3.35 (2.13,5.26)	< 0.001
Gender (male)	0.96 (0.59,1.56)	0.875
Age (years)	1.05 (1.03,1.07)	< 0.001
BMI (Kg/㎡)	0.89 (0.84,0.96)	0.001
HF (no)	4.9 (2.93,8.19)	< 0.001
IS (no)	2.28 (1.31,3.98)	0.004
CHD (no)	0.45 (0.28,0.71)	< 0.001
HBP (no)	1.32 (0.82,2.14)	0.253
DM (no)	1.5 (0.97,2.34)	0.071
COPD (no)	1.33 (0.57,3.09)	0.503
Smoke (no)	0.55 (0.35,0.87)	0.011
Drink (no)	0.44 (0.22,0.88)	0.02
CHA2DS2-VASc, (score)	1.41 (1.24,1.61)	< 0.001
SBP (mmHg)	0.9936 (0.9826,1.0047)	0.254
DBP (mmHg)	0.98 (0.97,1)	0.041
HR (beats per minute)	1.0042 (0.9881,1.0205)	0.613
WBC (×109/L)	0.88 (0.8,0.97)	0.009
Neutrophil (×109/L)	0.91 (0.83,1)	0.04

Lymphocyte (×109/L)	0.43 (0.28,0.67)	< 0.001
Monocyte (×109/L)	0.56 (0.2,1.56)	0.268
Hb (g/L)	0.97 (0.96,0.98)	< 0.001
PLT (×1012/L)	0.9925 (0.9885,0.9966)	< 0.001
CRP (mg/L)	1.08 (1,1.17)	0.043
Scr (umol/L)	1.01 (1.01,1.02)	< 0.001
Bun (mmol/L)	1.26 (1.16,1.37)	< 0.001
UA (umol/L)	1.0042 (1.0025,1.0059)	< 0.001
eGFR (ml/min)	0.98 (0.97,0.98)	< 0.001
TG (mmol/L)	0.63 (0.43,0.93)	0.02
TC (mmol/L)	0.71 (0.57,0.9)	0.005
HDL (mmol/L)	0.73 (0.31,1.72)	0.469
VLDL (mmol/L)	1.09 (0.51,2.33)	0.831
LDL (mmol/L)	0.69 (0.53,0.89)	0.004
FBG (mmol/L)	0.9995 (0.9029,1.1065)	0.993
HbA1c (%)	1.15 (0.92,1.42)	0.215
ALT (U/L)	1.0001 (0.9874,1.013)	0.982
AST (U/L)	0.9916 (0.985,0.9982)	0.013
TBIL (mmol/L)	1.05 (1.02,1.08)	< 0.001
IBIL (mmol/L)	1.23 (1.15,1.31)	< 0.001
ALB (g/L)	0.86 (0.82,0.91)	< 0.001

Sodium (mmol/L)	0.96 (0.91,1.02)	0.179
Potassium (mmol/L)	1.12 (0.65,1.91)	0.685
Chlorine (mmol/L)	0.98 (0.92,1.03)	0.4
CK (U/L)	0.9991 (0.9984,0.9997)	0.007
CTnT (pg/mL)	0.9995 (0.9992,0.9998)	0.003
NT-proBNP (pg/mL)	1.0001 (1.0001,1.0001)	< 0.001
D dimer (ug/mL)	1.34 (1.22,1.47)	< 0.001
LVEF (%)	0.96 (0.94,0.97)	< 0.001
LAD (mm)	1.09 (1.07,1.12)	< 0.001
LVDD (mm)	1.06 (1.04,1.09)	< 0.001

Notes: CHA2DS2-VASc is a scoring system for stroke risk assessment in patients with atrial fibrillation (congestive heart failure, hypertension, age ≥ 75 years, diabetes, prior stroke, vascular disease, age 65–74 years, and sex).

Abbreviations: VAP-1 =Vascular adhesion protein-1; AF = atrial fibrillation; BMI = Body Mass Index; HF = heart failure; IS = Ischemic stroke; CHD = coronary heart disease; HBP = high blood pressure; DM= diabetes mellitus; COPD = chronic obstructive pulmonary disease; SBP = systolic blood pressure; DBP = diastolic blood pressure; HR = heart rate; WBC = white blood cell; Hb = hemoglobin; PLT = platelet; CRP = C-reactive protein; Scr = serum creatinine; Bun = blood urea nitrogen; UA = serum uric acid; eGFR = estimated glomerular filtrationrate; TG = triglyceride; TC = total cholesterol; HDL = high-density lipoprotein cholesterol; VLDL = very low density lipoprotein cholesterol; LDL = low density lipoprotein cholesterol; FBG = fasting blood-glucose; HbA1c = Glycated hemoglobin A1c; ALT = serum glutamic pyruvic transaminase; AST = serum glutamic oxalacetic transaminase; TBil = total bilirubin; IBil = indirect bilirubin; ALB = serum albumin; CK = creatine kinase; CTnT = cardiac troponin T; NT-proBNP = N terminal brain natriuretic peptide precursor; LVEF = left ventricular ejection fraction; LAD = left atrial diameter; LVDD = left ventricular end-diastolic dimension; MACE = major adverse cardiovascular events.

Supplementary Table 7. Multivariate Cox regression analysis of risk factors associated with MACE in all patients.

Variable		Non-adjusted model HR (95% CI)	P value	model I HR (95% CI)	P value	model II HR (95% CI)	P value	model III HR (95% CI)	P value
**VAP-1(ng/mL)**	Q1 (≤729)	1(Ref)		1(Ref)		1(Ref)		1(Ref)	
	Q2 (729-1130)	1.35 (0.76~2.42)	0.31	0.92 (0.51~1.66)	0.772	0.92 (0.46~1.82)	0.809	0.77 (0.36~1.66)	0.507
	Q3 (≥1130)	2.17 (1.29~3.65)	0.004	1.45 (0.85~2.48)	0.169	0.76 (0.36~1.61)	0.476	1.02 (0.47~2.22)	0.964

Notes: data presented are HR and 95% CIs.

Non-adjusted Model: We did not adjust any covariants.

Model I: Adjusted for Gender, Age.

Model II: We adjusted for Model I + AF, CHA2DS2-VASc, HF, CHD, DM, Smoke, Drink, HR, Bun, UA, eGFR, TG, HDL, AST, TBIL, IBIL, NT-proBNP, D dimer, LVEF, LAD, LVDD.

Model III: We adjusted for Model II + BMI, SBP, DBP, HR, WBC, Hb, PLT, CRP, Scr, TC, LDL, FBG, ALT, ALB, Sodium, Potassium, CK, CTnT.

In each case, the model is not adjusted for the variable itself.

Abbreviations: VAP-1 =Vascular adhesion protein-1; MACE = major adverse cardiovascular events; AF= atrial fibrillation; HF = heart failure; CHD = coronary heart disease; DM= diabetes mellitus; HR = heart rate; Bun = blood urea nitrogen; UA = serum uric acid; eGFR = estimated glomerular filtrationrate; TG = triglyceride; HDL = high-density lipoprotein cholesterol; AST = serum glutamic oxalacetic transaminase; TBil = total bilirubin; IBil = indirect bilirubin; NT-proBNP = N terminal brain natriuretic peptide precursor; LVEF = left ventricular ejection fraction; LVDD = left ventricular end-diastolic dimension; BMI = Body Mass Index; HBP = high blood pressure; SBP = systolic blood pressure; DBP = diastolic blood pressure; HR = heart rate; WBC = white blood cell; Hb = hemoglobin; PLT = platelet; CRP = C-reactive protein; Scr = serum creatinine; TC = total cholesterol; LDL = low density lipoprotein cholesterol; FBG = fasting blood-glucose; ALT = serum glutamic pyruvic transaminase. ALB = serum albumin; CK = creatine kinase; CTnT = cardiac troponin T.
